# Effect of the Oxidation Process on Carrier Lifetime and on SF Defects of 4H SiC Thick Epilayer for Detection Applications

**DOI:** 10.3390/mi13071042

**Published:** 2022-06-30

**Authors:** Alessandro Meli, Annamaria Muoio, Riccardo Reitano, Enrico Sangregorio, Lucia Calcagno, Antonio Trotta, Miriam Parisi, Laura Meda, Francesco La Via

**Affiliations:** 1 Dipartimento di Fisica e Astronomia Ettore Majorana, Università degli Studi di Catania, Via S. Sofia 64, 95123 Catania, Italy; riccardo.reitano@ct.infn.it (R.R.); sangregorioenrico93@hotmail.it (E.S.); lucia.calcagno@ct.infn.it (L.C.); 2 CNR-IMM, Headquarter, Strada VIII 5, 95121 Catania, Italy; annamaria.muoio@imm.cnr.it; 3 ENI-MAFE, Via A. Pacinotti 4, 30175 Venezia, Italy; antonio.trotta@eni.com (A.T.); miriam.parisi@eni.com (M.P.); 4 ENI-Renewable Energy and Environmental R&D Center, Via G. Fauser 4, 28100 Novara, Italy; laura.meda@eni.com

**Keywords:** epitaxial growth, carrier lifetime, thermal oxidation process, 4H SiC, neutron detection, longitudinal optical phonon plasmon coupling (LOPC), photoluminescence, time resolved photoluminescence (TRPL), deep level transient spectroscopy (DLTS)

## Abstract

The aim of this work was a deep spectroscopical characterization of a thick 4H SiC epitaxial layer and a comparison of results between samples before and after a thermal oxidation process carried out at 1400 °C for 48 h. Through Raman and photoluminescence (PL) spectroscopies, the carrier lifetimes and the general status of the epilayer were evaluated. Time-resolved photoluminescence (TRPL) was used to estimate carrier lifetime over the entire 250 µm epilayer using different wavelengths to obtain information from different depths. Furthermore, an analysis of stacking fault defects was conducted through PL and Raman maps to evaluate how these defects could affect the carrier lifetime, in particular after the thermal oxidation process, in comparison with non-oxidated samples. This study shows that the oxidation process allows an improvement in the epitaxial layer performances in terms of carrier lifetime and diffusion length. These results were confirmed using deep level transient spectroscopy (DLTS) measurements evidencing a decrease in the Z_1/2_ centers, although the oxidation generated other types of defects, ON1 and ON2, which appeared to affect the carrier lifetime less than Z_1/2_ centers.

## 1. Introduction

The field of application of wide-band-gap solid-state detectors is expanding in those environments where radiation hardness is an indispensable feature. Among these fields of application are environments where high neutron flux is a problem, such as high flux neutron sources and thermonuclear fusion environments. Among the materials used for the detection application, silicon carbide (4H SiC polytype) could be considered the best choice, particularly where environmental conditions are critical for other materials, such as silicon [1,2], thanks to its radiation hardness. Nowadays, the best detectors used in this field are diamond-based, single-crystal diamond (SCD), with important performances, but the production capability of large-area wafers and the lower cost, with respect to the diamond, allow the use of SiC material. In a previous paper, the comparison between diamond and 4H SiC detectors with thicknesses of 100 micron was performed [3]. It has been noted that the efficiency of diamond detectors increases with increasing thickness because the probability of an interaction between the neutrons and the diamond substrate increases considerably. Then, also in the case of SiC, it is necessary to increase the thickness of the SiC layer to increase the efficiency of the detector. This can be done by using semi-insulating substrates that are 500 microns thick, or by trying to increase the thickness of the epitaxy. The first approach has been used in the past, but the performance of the detector was limited by the high defect densities of this material and its low carrier lifetime that reduce its charge collection [4]. Furthermore, this approach also shows some polarization effect and instability at high temperatures [5]. For this reason, a 250 micron epilayer was grown with a high growth rate process and a low doping level. In a previous paper [6], Kleppinger et al. characterized the defects in a 4H SiC Schottky barrier radiation detector fabricated on 250 μm epitaxial layers, highlighting the possibility of using SiC detectors in harsh environments. Epitaxy allows a high, precise control of thickness, homogeneity and doping concentration. In a previous paper, the epitaxial growth mechanism of this process using TCS (trichlorosilane) was described [7] and showed that it is possible to obtain very low doping levels, sharp interfaces, a low density point and extended defects. All these properties are important for neutron detectors. In fact, the low doping concentration is fundamental to using a lower depletion voltage for a thick detector. The low point defect density produces a large carrier diffusion length and then a good charge collection efficiency of a detector, while the low density of extended defects generates a high yield of the large-area detectors. In fact, from a previous paper it has been observed, through simulation with the FLUKA tool [8], that both thickness and area are important to increment a detector’s efficiency.

An in-depth study of the epilayer is critical to understand the performance of a future device. Some growth parameters influence the quality of the final layer. Among all, the conditions of growth rate and the Si/C ratio play an important role in the defect formation and annihilation. Stacking fault (SF) defects are commonly present in the epitaxial layers. In general, as reported by Kimoto [9] and La Via [10], they are obtained from basal plane dislocation (BPD) propagation from the substrate into the epilayer, but they also can appear directly in the epilayer during growth. Previous studies evidenced the influence of this SF defect on the increment in the recombination current, and this recombination is dependent on the levels introduced in the band gap by the different kinds of SF defects [11].

One of the main parameters that influence the performances of a device is the carrier lifetime, and this parameter could be affected by various kinds of defects.

Carrier lifetimes in n-type 4H SiC have been intensively investigated in recent years [12,13,14]. Despite the relatively long carrier lifetime obtained in previous studies [15,16], it is possible to improve this parameter by identifying and reducing the causes that lead to the reduction in the lifetime, therefore the killer defects.

Many types of defects can adversely affect the carrier lifetime, in particular the Z _1/2,_ which is considered the dominant lifetime killer, and it is a common intrinsic defect in this material. In previous studies, the influence of growth parameters during the CVD process was evaluated [17,18,19]. Moreover, a direct correlation between Z_1/2_ centers and carrier lifetime was defined through low-energy electron irradiation that allows a displacement of carbon atoms, highlighting that this type of defect could be a carbon vacancy or carbon interstitial [12,20]. There are some processes that allow reduction or elimination as either carbon ion implantation [21,22] or thermal oxidation [23,24].

The Z _1/2_ center, which is located at 0.65 eV below the conduction band edge, is now recognized as the dominant lifetime killer, at least, in n-type 4H SiC. As described in the literature, thermal oxidation or carbon ion implantation followed by high-temperature annealing was used to eliminate or reduce the Z_1/2_ center concentration. On the contrary, new depth levels, ON1 (E_C_ −0.84 eV) and ON2 (E_C_ −1.1 eV), are detected after thermal oxidation or C^+^ implantation, followed by Ar annealing [21], which could be related to interstitials diffusing from the SiO_2_/SiC interface (oxidation), or from the implanted region (C^+^ implant). Although the effects of the centers ON1 and ON2 on the lifetime are negligible with respect to the effects of the Z_1/2_ centers [25], they may have an effect, not yet well identified, on the carrier lifetime but still less than that of the Z_1/2_ centers. The decrease in carrier lifetime is attributed not only to Z_1/2,_ but also surface and interface recombination and extended defects as staking faults [26,27].

In a previous paper, the effect of different stacking fault defects was evaluated [28], and a decrement of carrier lifetime, on and around the SFs, was observed. In the present work, the carrier lifetime in n-type 4H SiC was further improved by employing a high-temperature and long oxidation process, 1400 °C for 48 h, for a very thick epilayer. In order to obtain this information, PL and LOPC Raman spectroscopies were used. Time-resolved photoluminescence was used to evaluate the carrier lifetime across the entire epilayer, using different wavelengths of the source. The decrease in Z_1/2_ centers, which led to an increase in the carrier lifetime after thermal oxidation process, was evaluated using DLTS measurement. Then, a study of the influence of different kinds of SF defects on the carrier lifetime was evaluated after the oxidation process and compared with a non-oxidated sample.

## 2. Materials and Methods

A 4H SiC (0001), n-type, silicon face and an off axis of 4° was used as a substrate. A thick (250 µm) and low-doped (5 × 10^13^/cm^3^) epitaxial layer was grown using the chemical vapor deposition process in a horizontal hot-wall reactor (LPE PE106).

The oxidation process was conducted after the deep cleaning of the sample with acetone, isopropanol and methanol, each in an ultrasonic bath for five minutes. After an RCA process (20 min), cleaning with piranha solution and HF was performed. Furnace ramp up and ramp down was performed at 8 °C/min in Ar atmosphere, and then pure dry O_2_ oxidation (0.5 L/min) at 1400 °C for 48 h was conducted.

Micro Raman and photoluminescence maps were acquired using an HR800 integrated system Horiba Jobin Yvon [29] in the back scattering configuration. The accuracy of the instrument at room temperature (±1 °C) is about ±0.2 cm^−1^. The spatial resolution achievable with the motorized stage is 0.5 µm and is also used for spot laser definition. A He-Cd Laser with a wavelength of 325 nm was used for these measurements. The laser power was changed from 0.15 to 15 mW using different filters. A x40 objective was used to focus the laser. The diameter of the laser spot was about 9 µm. This value was extracted following the Raman signal across a metal line previously realized using photolithography. Each Raman spectrum was collected with an acquisition time of about 1 s. For the lowest laser power (0.15 mW), the acquisition time was increased (12 s) in order to obtain an appreciable signal.

Time-resolved photoluminescence measurement was conducted using a 5 µs pulsed lamp. The wavelengths of the polychromatic emission pulse were selected with a low straylight double monochromator before reaching the sample. The luminescence decay was recorded with a photomultiplier and using a decay-by-decay technique, with a time window of 0.1 ms.

Deep level transient spectroscopy (DLTS) measurements were carried out by means of a Sula Technologies, Ashland in Oregon (USA), double boxcar spectrometer with exponential correlator measurements in the temperature range 100–750 K by using rate windows in the range 2–200 s^−1^.

## 3. Results and Discussion

The status of epitaxy evaluation was performed through room temperature photoluminescence to define the presence of SF defects. A comparison between the 4H SiC thick sample before and after the thermal oxidation process was evaluated.

**Figure 1:**
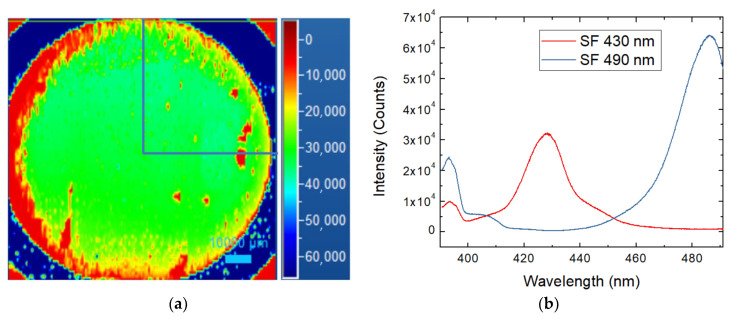
Photoluminescence analysis of the 4H SiC 4-inch wafer (**a**) and the common stacking fault defect on the highlighted quarter of the wafer (**b**) located mainly on the edge side.

Photoluminescence spectroscopy allows the detection and analysis of different crystalline defects inside the epitaxial layer. These defects act as recombination centers and, in some cases, we could define, in particular for bar-shaped SFs, the depth at which the defect is generated, knowing its length and the step flow direction. Therefore, it is possible to calculate if the SF defects start from the epilayer/substrate interface in the basal planes [30]. As shown in Figure 1a, a photoluminescence map of the entire 4-inch wafer was performed, evidencing the presence of some stacking fault defects, mostly located on the left edge of the wafer, and this observation was confirmed by the wavelengths of the peaks obtained, specific for this type of stacking fault. This position is generally observed in thick epilayer growth and is due to the fact that the step flow is going from the left of the wafer to the right and in the left border, the growth is on-axis, and the probability of stacking fault formation is much higher than in the case of step flow.

Only a quarter of the wafer was used for the oxidation process, in particular the area delimited by the blue lines in Figure 1a. In this quarter, just two kinds of SFs are present, at 430 and 490 nm of wavelength (Figure 1b), but we cannot see the presence of carbon vacancies directly.

The most common defects that can strongly affect the carrier lifetime are not only the carbon vacancies, defined as Z_1/2_ centers, but also extended defects, such as stacking faults. In 4H SiC epilayers, the carrier lifetimes can be limited by other recombination paths, such as recombination near the epilayer/substrate interface and surface recombination [31,32]. The thermal oxidation process, as mentioned before, leads to an increment in carrier lifetime thanks to the Z_1/2_ centers’ decrement confirmed using DLTS measurements.

**Figure 2:**
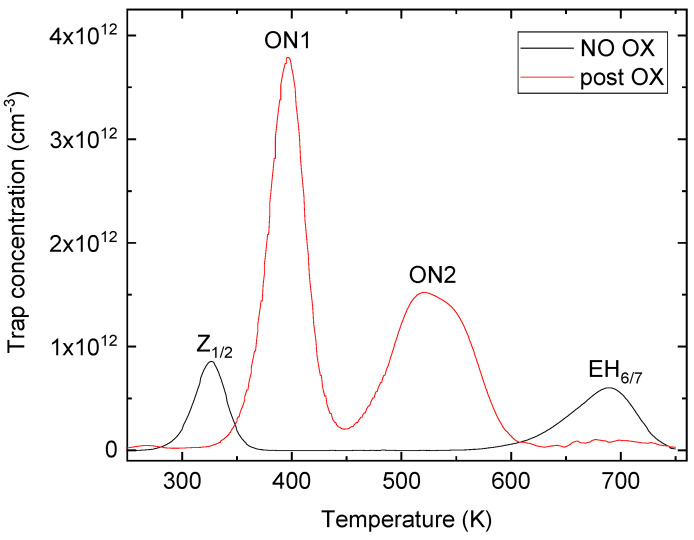
Deep level transient spectroscopy (DLTS) measurements on non-oxidated (black line) and post-oxidated (red line) samples with the appearance of new ON1 and ON2 centers.

Figure 2 shows the DLTS spectra acquired at a rate window of 4.65 s^−1^ of the non-oxidized sample (black line) and after thermal oxidation (red line). The dominant levels in the no-ox sample are Z_1/2_ (Ec −0.62 eV) and EH_6/7_ (Ec −1.5 eV); the energy levels were determined, as usual, using an Arrhenius plot of measurements performed at different rate windows. The concentration of these levels was 0.8 × 10^12^ cm^−3^ and 0.6 × 10^12^ cm^−3^, as reported in the literature [6] for this thick epilayer, and also, the small capture cross section of 10^−15^–10^−16^ cm^2^ indicated that these defects were mostly single atoms, rather than clusters. Despite this, Z_1/2_ centers could influence lifetime, but these two levels disappeared after oxidation, while two new traps, ON1 (Ec −0.78 eV) and ON2 (Ec −1.1 eV), were generated near the surface region, but could be extended further across the entire epilayer using a higher temperature for a long time [33]. The concentration of ON1 and ON2 levels was 3.8 × 10^12^ cm^−3^ and 1.5 × 10^12^ cm^−3^, respectively. In this case, an oxidation process at 1400 °C for 48 h was performed, and despite the fact that annealing with Ar as the post-oxidation process was not conducted, the Z_1/2_ centers were eliminated. However, ON1 and ON2 centers appeared, and the exact structure and formation reaction of these centers was not known, but the concentration decrease with the depth and their influence on carrier lifetime was negligible compared to Z_1/2_ centers [34].

**Figure 3:**
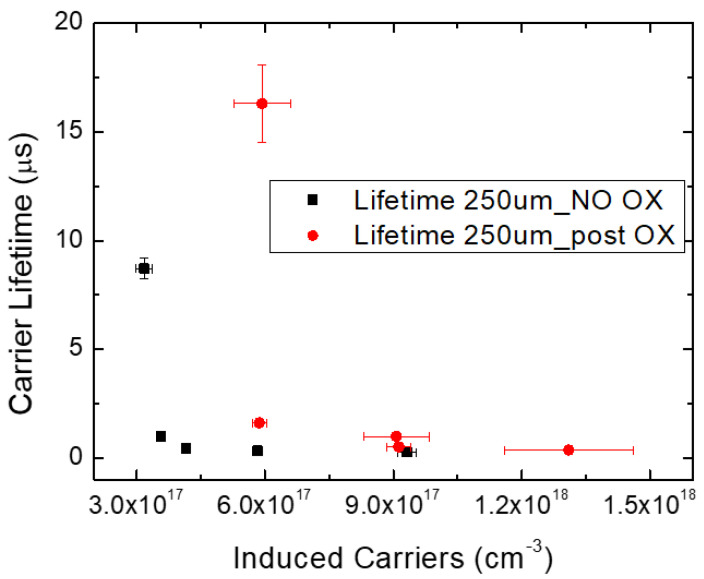
Carrier lifetime as a function of induced carriers, before (NO Ox) and after (Post OX) the oxidation process.

As mentioned before, the oxidation process allows a consistent decrement in Z_1/2_ centers, thanks to the reduction in carbon vacancies, leading to carrier lifetime increment into the epilayer. This increment was appreciable using Raman spectroscopy, focusing the attention on the longitudinal optical peak shift, and is shown in Figure 3. The longitudinal optical phonon–plasmon coupling mode (LOPC) was used to extract carrier density and carrier lifetime. This method is widely described in the literature [35,36,37] and usable for low-doped samples [38].

In Figure 3, the carrier lifetime as a function of the induced carriers using different laser powers during the Raman analysis is shown, observing an increment in carrier lifetime as the induced carriers decrease. The red points are the data obtained after the oxidation process, and it seems that there was an increment in the carrier lifetime, more visible at lower induced carrier values. This was due to the fact that if we have an injection level under a value of 10^18^ cm^−3^, the Auger recombination (AR) can be neglected [13]. However, as reported in the literature [39], monomolecular, bimolecular and Auger recombination coefficients can influence carrier lifetime. Auger recombination (AR) became important when the doping or the excess carrier density became higher. Moreover, there is a temperature dependence of the Auger recombination coefficient (γ_3_^eeh^), specifically, the higher the temperature is, the lower the Auger recombination coefficient is. Finally, this AR coefficient can directly be derived from the relationship γ_3_^eeh^ = [τN^2^_D_]^−1^, where τ is the carrier lifetime and N_D_ is the carrier concentration; the oxidated sample shows a lower AR coefficient than a non-oxidated sample, hence a minor contribution of Auger recombination at high carrier density values favors a higher carrier lifetime.

**Figure 4:**
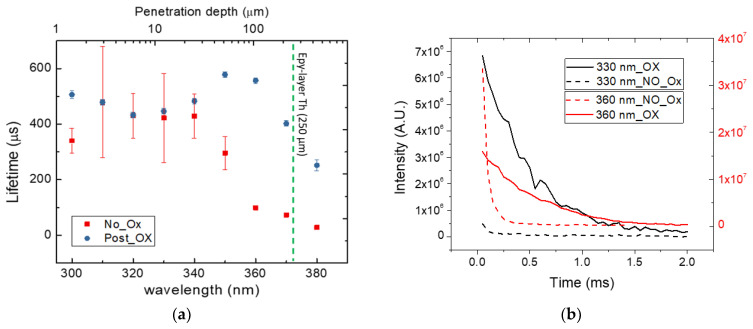
Time-resolved photoluminescence measurements at different source wavelengths in order to obtain information from different depths, before and after oxidation process (**a**), and the decay curve obtained by this measure at 330 and 360 nm for both samples (**b**).

These measures were conducted in SF-free areas and allow us to understand the direct increment in the carrier lifetime after the oxidation process in the high injection regime. This hypothesis is also supported by time-resolved photoluminescence (TRPL) measurements to evaluate the carrier lifetime in a low injection regime using different wavelengths to study different penetration depths. The comparison between oxidated (Post OX) and non-oxidated (NO Ox) samples is shown in Figure 4.

The wavelengths used for the measurements were ranging from 300 to 380 nm with penetration lengths between 4 and 600 µm. The last point at 380 nm is referred to as a deep penetration depth, because the greater the wavelength, the deeper the beam penetrates, hence we were going through the entire epitaxial layer until we reached the top of the substrate. For this reason, the carrier lifetime value was lower than the measurements at lower wavelengths. Due to the large spot of the lamp, the signal obtained was averaged over the analyzed area, but the trend was clear and showed an increment in carrier lifetime for the oxidated sample compared with the non-oxidated sample, especially close to the substrate interface. The different decays at the same wavelength are shown in Figure 4b, where a lower decay and then a higher carrier lifetime value can be observed for Post Ox samples. This difference was particularly evident at higher wavelengths or higher penetration depths. It should be remembered that the measurement of the lifetime was strictly linked to the measurement technique used and, in this case, the values obtained with TRPL were greater, as we were in a low injection regime compared to the measurements made with Raman.

Through this analysis, we confirmed the possibility of increment in carrier lifetime after an oxidation process, which also leads to an increase in the diffusion length, obtained by L_D_ = √Dτ, where *D* is the diffusion coefficient and τ is the lifetime of the excited carrier. Considering a mobility value of 900 cm^2^/Vs and a doping concentration of about 10^14^ cm^−3^ (low injection regime), an increment in diffusion length was observed from 712 µm (NO Ox) to 911 µm (Post Ox).

As stated above, carrier lifetime evaluation was conducted far from any SF defects, but they were present in the epilayer, even if in low percentages, and could affect the performances of the devices by increasing the leakage current and decreasing the carrier lifetime. For this reason, a study of the influence of different SF defects on carrier lifetime was performed to estimate if the oxidation process improves the lifetime on and around these defects.

**Figure 5:**
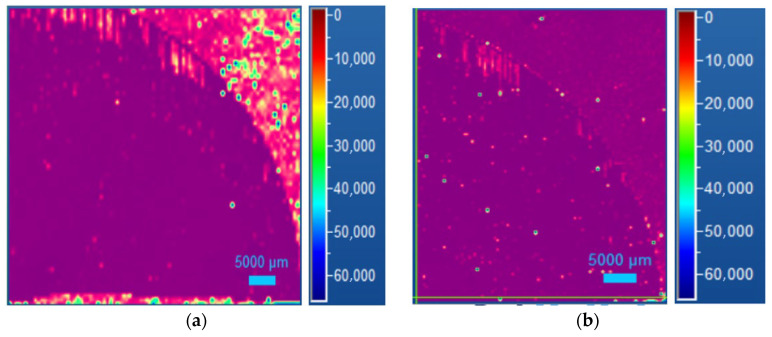
Photoluminescence map of the same quarter of the 4H SiC wafer highlighted in Figure 1a, before (**a**) and after (**b**) oxidation process.

From both maps in Figure 5 showing before and after the oxidation process, it is possible to evaluate the same SF defects in the same position. However, from a thorough analysis, we observed different behavior after the oxidation process for the defects at 490 nm (2.53 eV), compared to the defects at 430 nm (2.88 eV).

**Figure 6:**
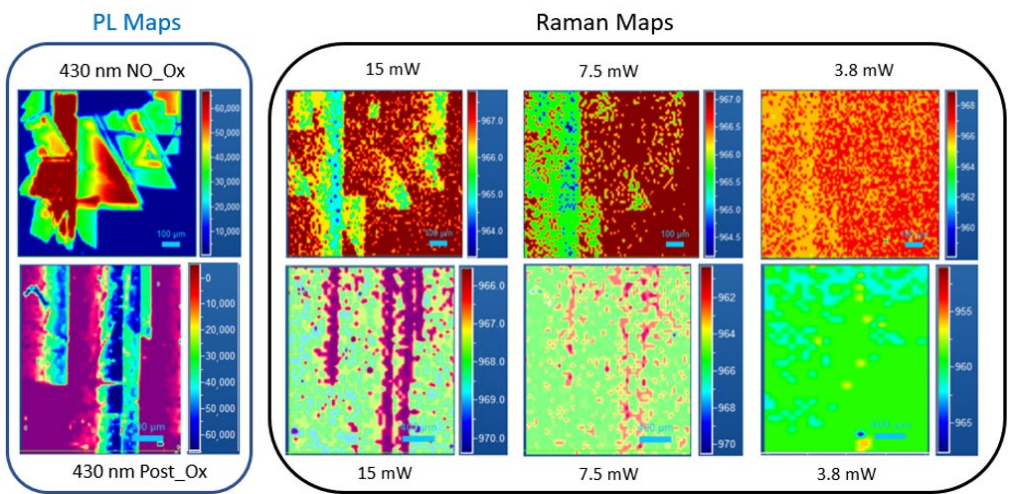
PL and Raman maps of an isolated defect at 430 nm No_Ox (upper strip) and Post OX (lower strip) for different induced carrier values related to the laser power used (15–7.5–3.8 mW, respectively) following the LO peak position.

Both defects show a lower carrier lifetime than the values obtained in the SF-free area and the LO shift difference between IN and OUT SF decreases when the laser power used was decreased, obtaining the same carrier lifetime values for a particular power of the laser. However, this decrement differs depending on the type of defect. As shown in Figure 6, for a 430 nm defect, the Raman maps obtained following the LO peak position show a uniform contrast at 3.8 mW, which are the same results of the non-oxidated sample for the same defect.

Instead, for the 490 nm defect, it was observed that when using 15 mW of laser power, the data acquired on the SF defect showed a strong Raman shift reduction, suggesting a trapping of free carriers. Before the oxidation process, both defects analyzed were observed, and under 3.8 mW, there are no differences between IN and OUT SF spectra; instead, it was different for the oxidated sample, where a larger contrast was observed.

**Figure 7:**
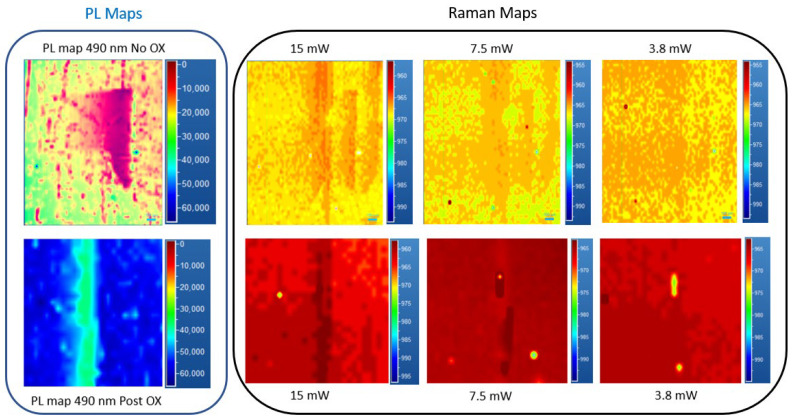
PL and Raman maps of an isolated defect at 490 nm No_Ox (upper strip) and Post OX (lower strip) for different induced carrier values related to the laser power used (15–7.5–3.8 mW, respectively) following the LO peak position.

**Figure 8:**
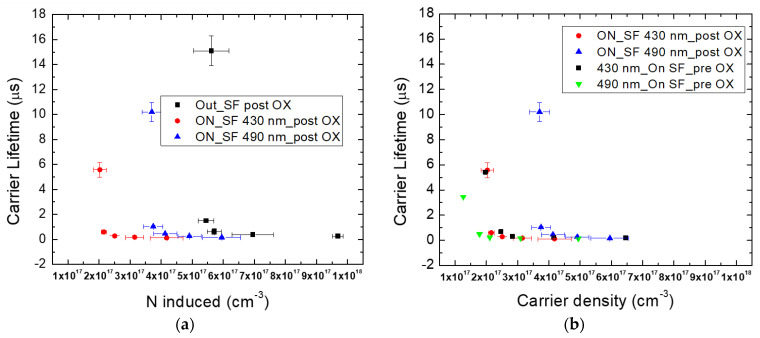
Carrier lifetime as a function of carrier induced. Comparison between SF-free area and defective zone (**a**); comparison between the same defects (430–490 nm) before and after oxidation process (**b**).

Figure 7 shows, as in the previous figure obtained for 430 nm SF, PL and Raman maps of a 490 nm SF defect related to the laser power used. It is evident that, moving to a low injection region, the influence of the SF defect on carrier lifetime decreased. This effect is more evident for the oxidated sample, where a minor influence on the part of the SF was observed, as is shown in Figure 6 and Figure 7. This suggests that the same values of carrier induced, and the lifetime IN and OUT, of the defect (in proximity) are obtained when low laser power is used. This difference is more evident in Figure 8.

From these results, it is evident that the oxidation process leads to an improvement in the carrier lifetime. Moreover, not all the stacking fault defects undergo the same improvement. Considering the lifetime, the 490 nm defect is much more influenced by the oxidation process than the 430 nm. In fact, the 490 nm SF shows a large increase in the carrier lifetime at induced carriers of about 4 × 10^17^/cm^−3^ after the oxidation process; before this process, the increase in carrier lifetime is around 1.5 × 10^17^/cm^3^ (Figure 8b). Instead, in the case of the 430 nm SF, the increase in the carrier lifetime remains the same, at the same induced carrier conditions.

Despite this different behavior of the defects, even after oxidation, they lead to a decrease in the lifetime, compared to the SF-free areas (Figure 8a), leading to a recombination or trapping of the free carriers. In fact, it is clearly shown in Figure 8a that the induced carriers, at which a large increase in the carrier lifetime is observed, increases with respect to the region without SFs both for the 430 and the 490 nm SFs. It seems that the 490 nm SF is less efficient in the reduction in the carrier lifetime, with respect to the 430 nm.

## 4. Conclusions

A spectroscopical characterization of a 250 µm thick epitaxial layer was performed using Raman spectroscopy in longitudinal optical phonon–plasmon coupling mode for high carrier densities and time-resolved photoluminescence for low carrier densities. This material will be used for the fabrication of devices for neutron detection in harsh environments and, for this application, it is important to have a consistent volume to improve the detector’s efficiency. Not only the volume but also the quality is fundamental for good reliability, and the carrier lifetime plays an important role because it can strongly affect the performances of the devices. An oxidation process was conducted evaluating an increment in carrier lifetime, especially going towards a low injection regime. This phenomenon is also supported by the low impact of the SFs on carrier lifetime at low carrier injection, which became important for detector applications, where particles induce a very low carrier injection. Furthermore, the oxidation process influences, in different ways, the kinds of SFs analyzed, where the 490 nm defects increase the carrier lifetime value compared to the value of the non-oxidated sample, while the 430 nm does not seem to be affected by the oxidation process. Finally, the development of epitaxial growth and the possibility of increasing the carrier lifetime through the oxidation process allow performance improvement compared to the previous detectors studied with thicknesses of 100 µm.
